# Shared Experiences of Resilience Amongst Couples Where One Partner Is Living With Dementia—A Grounded Theory Study

**DOI:** 10.3389/fmed.2020.00219

**Published:** 2020-06-09

**Authors:** Lucy Conway, Emma Wolverson, Chris Clarke

**Affiliations:** ^1^Tees, Esk and Wear Valleys, NHS Foundation Trust, Darlington, United Kingdom; ^2^Faculty of Health Sciences, The University of Hull, Kingston upon Hull, United Kingdom

**Keywords:** resilience, dementia, couplehood, qualitative, well-being

## Abstract

Resilience is a concept which may help explain how older people are able to live well with dementia. Existing resilience research in dementia focuses on the caregiver and relatively little is known about how dyads (person with dementia and care partner) experience resilience. Using constructivist grounded theory, this qualitative study aimed to develop a theory of shared resilience amongst couples where one partner is living with dementia. Interviews were conducted with 12 dyads (*n* = 24) to explore their shared understanding of resilience, what helps to develop and maintain their resilience and how resilience shapes their relationship and mutual well-being. Findings indicate that resilience was experienced as continuing with a “normal” life as a couple notwithstanding the impact of dementia. This is in contrast to models of resilience which emphasize bouncing back or flourishing in the face of adversity. Instead, couples described a shared resilience that enabled them to maintain their couplehood, a sense of togetherness and reciprocity in their relationship, which in turn provided a further source of resilience. Findings emphasize the importance of dyadic research in developing a clearer understanding of the experience of living well with dementia. Interventions aimed at building resilience should engage dyads to consider how the couple's shared resilience can be maintained and enhanced.

## Introduction

Resilience is a concept that may help explain why some people are able to continue to live meaningful lives despite facing adversities associated with dementia (1). Narratives of resilience promote a strength-based approach to dementia that moves beyond dominant discourses of loss and deficit (2). Such an approach furthers our understanding of what it means to live well with dementia (1) and as such is a vital way to empower people living with dementia (PLwD).

Lepore and Revenson (3) outlined three dimensions from which the construct of resilience can be understood: “resistant resilience,” remaining strong in the face of adversity; “recovery resilience,” bouncing back to previous functioning after facing adversity; and finally, “reconfiguration resilience,” going through a period of growth after adversity. This final form of resilience is separate to post-traumatic growth although research often fails to distinguish between them (4).

To date, the majority of research into resilience in dementia has focused on caregivers, with findings indicating that resilience can increase both physical and mental well-being by reducing anxiety, depression and by fostering coping (5). High levels of caregiver resilience benefit PLwD by reducing rates of care recipient institutionalization and even death (6). To date, little research has directly explored resilience with PLwD, possibly due to an assumption that PLwD cannot live well (7) and/or may not be able to sustain or develop resilience. However, two recent qualitative studies shed more light on the potential dimensions of resilience in living with dementia. Williamson and Paslawski (8) interviewed seven people with dementia who described resilience as process of maintenance, the ability to adapt what they did to keep life as normal as possible after diagnosis. Clarke and Bailey (9) highlight the importance of a sense of belonging in a social and physical space in enabling resilience for people with dementia.

An important way to further our understanding of resilience in dementia is to take a dyadic approach. This is important given that dementia affects both members of a couple individually while also potentially influencing their couplehood. Couplehood has been defined as a mutual sense of togetherness and reciprocity in the dyad, which provides insight into how couples live together with the challenges that dementia brings (10). Wadham et al. (11) suggest that a shared sense of resilience in couples may enable them to achieve and maintain a sense of couplehood.

A significant barrier to furthering our understanding of resilience with respect to living with dementia is that there remain few theoretical frameworks from which research can be based. At present, within dementia, definitions of resilience vary widely and include: adjusting to adversity, a personal attribute, well-being and inner strength, level of burden and sufficient social support (12). Proposed definitions of resilience vary between professionals and caregivers, with caregivers focusing more on social support and the relationship with PLwD, whilst professionals prioritize maintaining quality of life and coping (13). Whilst prominent frameworks of resilience exist [for example (14)] and have been cited in relation to caregivers within the dementia literature [for example (15)], they are not specific to dementia and do not incorporate relational factors.

Given the need for a theoretical framework of resilience in dementia, particularly in relation to the perspective of PLwD and the dyad, this study aimed to generate a theory of shared resilience using a constructivist grounded theory approach. This study therefore aimed to explore what resilience means in the context of couplehood in dementia, how dyads experience a shared sense of resilience, how they develop and maintain resilience and how this impacts upon their relationship.

## Methods

### Design

This qualitative study used Charmaz's approach to constructivist grounded theory (16) to guide sampling, data collection, and data analysis. Grounded theory is a research method intended to develop a theoretical explanation of a social phenomenon that is “grounded in” naturalistic data.

### Sample

Within constructivist grounded theory there are no agreed standard sample sizes; the aim of the data collection being to reach data saturation—when further collection of data will not lead to additional information related to the research question (16). Guest et al. (17) suggest this can be achieved with between 6 and 12 interviews. In accordance with grounded theory, initially purposeful sampling was used to select people who were likely to have had a broad range of experiences but who considered themselves to be resilient. Subsequent participants were selected using theoretical sampling, whereby initial data analysis informed subsequent decisions regarding what data would be collected next and from whom. Participants were recruited from across Yorkshire (UK) via a local NHS Foundation Trust, memory cafés run by the Alzheimer's Society and the Join Dementia Research database. Table 1 provides an overview of the inclusion and exclusion criteria for the research.

**Table 1:** Participant inclusion and exclusion criteria.

**Inclusion criteria**	**Exclusion criteria**
One member of the dyad to be living with dementia and aged over 65 years.	Dyads with a diagnosis of young onset dementia (YOD). Research indicates that the experiences of people with YOD may differ from people diagnosed in later life (18). People who had received their diagnosis <3 months previously. Research (19) indicates that it takes around 3 months to adjust to a diagnosis and this study was focused on the experience of living with dementia rather than that of receiving a diagnosis.
The other member of the dyad to be over 18, a spouse or partner of the person with dementia and to self-identify as their caregiver.	
The dyad is living together within the community, as moving to residential care can significantly change roles and the relationship (20).	
Both participants have capacity to give consent to involvement in the study.	

Twelve dyads took part in total. Ten males and two females were diagnosed with dementia, with a range of diagnoses: mixed (*n* = 5), Alzheimer's Dementia (*n* = 4), Vascular Dementia (*n* = 1), Lewy Body Dementia (*n* = 1), and unknown subtype (*n* = 1). Participants received their diagnosis between 3 months and 6 years ago (mean = 2.9 years). The age of PLwD ranged from 67 to 89 (mean = 75.3). Of the caregivers taking part in the research, 10 were female and 2 were male, ranging in age from 53 to 87 (mean = 72.3). Eleven of the dyads interviewed were married, ranging from 22 to 66 years (mean = 46.2 years); 1 dyad was unmarried, but had been in a relationship for 10 years. All participants interviewed were White British. Table 2 describes characteristics and pseudonyms of the participants.

**Table 2:** Participant characteristics.

**Dyad surname pseudonym**	**Years together**	**Time since diagnosis**
Jones	49	2 years
Davies	28	1 year
Roberts	51	1 year
Brown	66	6 years
Smith	52	5 years
Evans	24	2 years
Williams	10	3 years
Thompson	50	3 years
Green	59	3 years
Taylor	22	3 months
Wood	60	2 years
Edwards	47	6 years

### Ethics and Consent

The London—Riverside Research Ethics Committee and the UK Health Research Authority (Approval No. 17/LO/1121) granted ethical approval prior to commencing the study. Each dyad was provided with verbal and written information about the study prior to gaining consent. An assessment of capacity to give informed consent was made by the lead researcher (LC), assessed by ensuring both members of the dyad were able to understand and retain information about the study, weigh up the information in order to make a decision about participating in the study and then communicate this decision to the researcher. Both members of the dyad were asked to complete written consent forms. One dyad who expressed interest were excluded because one member was unable to provide informed consent to take part.

### Data Collection

All dyads were interviewed by choice in their home once. Each dyad completed a demographic questionnaire. The interviews then commenced, with the researcher interviewing the dyad together, allowing an opportunity for shared meanings to be developed and new knowledge to be generated between the dyad (21). Dyads were asked about their understanding of resilience, what had helped to develop and maintain their resilience as a couple and what impact their resilience had had on their relationship and shared well-being. Consistent with a grounded theory approach, theoretical sampling was used to help clarify and address gaps in any emergent theory (16). This centered on adding and adapting interview questions over the course of data collection on the basis of initial coding and constant comparative analysis. Both members of each dyad participated in the interviews; however, participation was not always even as PLwD led some interviews, whilst in others it was their partner who led. Interviews ranged in length from 37 to 75 min (mean = 56 min). Dyads were invited to provide further information following the interviews via email or telephone, with one dyad responding. In accordance with Charmaz (22), saturation was reached as further data collection did not give further detail to the categories or theory. Consideration of data saturation, time constraints, and management of data therefore determined the sample size of 12 dyads.

### Analysis

Each interview was transcribed verbatim prior to the next interview, allowing for constant comparative analysis. Analysis of data was carried out referring to recommendations made by Charmaz (16). Coding of the transcripts began with initial coding, in which data was coded line by line and key quotes and ideas were identified and highlighted within the margin of the transcriptions. We then categorized the initial codes that were most frequently mentioned and also most meaningful, using focused coding to analyze larger sections of data. Focused codes were then used for theoretical coding in which the categories developed by focused coding were compared, developing overarching themes in which interrelationships between them were conceptualized and mapped, forming an emergent theory of shared resilience. This was an iterative process; we revisited earlier codes, comparing these repeatedly to emerging overarching themes. We also consulted previous research and literature examining resilience for similarities and differences and to see how similar findings had been categorized and grouped in order to aid the development of the theory. Quotes from participants were integrated into the narrative of our findings, selected to illustrate and represent the experiences of participants and the emergent theory (23).

## Results

Four main themes emerged from the data: Understanding Resilience, Shared Resilience, Developing Resilience, and Resources—Support and Stability. Each theme is described with illustrative quotes from interviews and an emergent theory is then presented to describe the development and maintenance of shared resilience.

### Understanding Resilience

Participants initially struggled to define resilience, with one caregiver stating “I don't know really, what exactly does resilience mean?” (Mrs. Smith—caregiver), whilst Mr. Taylor described it as “the ‘in’ word that's come from somewhere” (Mr. Taylor—PLwD). Difficulties defining the term appeared to stem in part from the construct being so deeply embedded in participants' everyday lives. The majority of couples framed resilience as the importance of being able to continue living the life they had before the dementia, both individually and together. For example, Mr. Smith described resilience as “maintaining what I've been used to doing, all be it not as well” (Mr. Smith—PLwD). For couples, feeling resilient related to continuing activities together. Continuing their shared activities, confirmed for them that they were coping, able to “just lead a normal life” (Mr. Jones—PLwD) and “keeping a hold on, the sort of life that we used to have, the values of that life, erm and not letting dementia win” (Mrs. Evans—caregiver). As such, ideas of resilience and well-being were often enmeshed for couples -“I'd assumed that we're, we are talking about our happiness as a couple, cos I think, we're saying we've got plenty of resilience, if we hadn't we wouldn't be very happy” (Mrs. Evans—caregiver).

Resilience was therefore a process of maintenance and continuity of a shared life, but this required active effort and agency. Couples described adopting two positions in their attitude and approach to living with dementia, one was the decision to remain positive and the second was a decision to fight. Remaining positive was not necessarily shared by both partners but instead tended to be “held” by one partner for the benefit of the other, with one caregiver stating “I'm a great believer in looking on the bright side of things” (Mrs. Brown—caregiver). A sense of humor was frequently referred to by couples as helping maintain a positive outlook; “there's always been a sense of humor” (Mr. Williams—PLwD). The decision to fight the dementia was always shared between the couple; Mrs. Roberts describes their approach to “fighting against it (dementia), fighting against what it's making him into sort of thing, and making our lives” (Mrs. Roberts—caregiver).

### Shared Resilience

A theme of shared resilience and a resilient togetherness dominated all the interviews; “there is an absolute determination that dementia is not going to come between us” (Mrs. Evans—caregiver). Couples described how they had grown closer since the dementia, with one caregiver stating that they “are now more of a couple than two individuals getting on with life” (Mrs. Roberts—caregiver), whilst another caregiver spoke about how since dementia the couple “live more harmoniously than we did before” (Mr. Edwards—caregiver).

Couples described how their relationship and sense of togetherness enabled them to develop and maintain resilience, but also how their resilience improved the quality of their relationship, enabling them to continue living the same kind of life; “resilience is trying to strengthen our relationship if possible and it's something that I've been doing… so that we can continue to live together through this diagnosis” (Mr. Edwards—caregiver).

For some couples, time spent in the relationship was important for the development of their shared resilience, with a caregiver stating “we've been together nearly 50 years, would I feel like this if I'd only been together 7,8,10?” (Mrs. Jones—caregiver). For others it was more about the quality of relationship they felt they had; the Williams who had been in a relationship for 10 years, the shortest amount of time in comparison to the other couples interviewed, disagreed, stating, “we're comfortable, if we'd have been for 50 years we couldn't be more comfortable with each other” (Ms. Williams—caregiver).

An important factor in the maintenance of shared resilience was demonstrating and sharing acts of love, with a caregiver stating “when things get a bit rough we give each other a kiss, it's amazing how er how that helps” (Mrs. Evans—caregiver). Another caregiver mentioned that she appreciated her husband buying her flowers every week, despite his age, dementia, and arthritis. A further way in which shared resilience was maintained was by talking together about the dementia, talking through and solving the problems they faced. However, discussion about the dementia had to be limited, with one participant living with dementia stating that they “don't talk about it every day” (Mrs. Edwards—PLwD). Not talking about dementia every day allowed priorities to be given to other things in their life. Couples spoke of actively shifting the perspective on dementia and relegating it to the background in terms of their values, priorities and goals, ensuring that “Alzheimer's is something that is a shadow” (Mr. Edwards—caregiver).

For both members of the couple, retaining a sense of independence was important in providing respite and an opportunity for both to recharge their batteries. For PLwD it was important that their diagnosis did not negatively impact on their partner, with one person saying “I don't hold her back from doing things” (Mr. Brown—PLwD). By doing this it was hoped that their relationship would be able to continue in the same positive way, as Mr. Taylor explained not doing so “would destroy her anyway and destroy our relationship” (Mr. Taylor—PLwD).

### Developing Resilience

When seeking to explore where resilience came from, responses varied widely both within and between couples. Some felt their resilience was part of who they were as individuals and therefore a personality trait not affected by dementia, with one person stating “if you're that way inclined I think it goes straight through your life” (Mr. Green—PLwD). Others felt that their resilience developed due to previous experiences, particularly adversities they faced during childhood, “I think the start of your life” (Mrs. Wood—PLwD). Participants also described how shared, difficult experiences since being a couple had developed their shared resilience and been important in developing the fighting approach that couples described as important in facing adversities associated with dementia; “we've had to face up to problems in life like that and that's the approach” (Mr. Wood—caregiver).

The development of resilience (both individual and shared) was experienced as a continual process, growing over time with age; “maybe it's just something that we've learnt as we've gone on… I think it probably has grown over the years” (Mrs. Taylor—caregiver). It was also a direct result of living with dementia “as a result of the diagnosis and that increased dependence on each of us erm, I think that has strengthened the relationship and provided that resilience” (Mr. Edwards—caregiver).

Some couples stated that, over the years, they have not always been resilient, with their resilience varying depending on “what you've got to cope with erm, as to whether then you can be resilient enough to cope with it” (Mr. Taylor—PLwD). It was important for couples to accept that “you can't be strong all of the time” (Mrs. Green—caregiver), and that “sometimes you just feel knocked down and vulnerable, and I think you've just got to accept that” (Mrs. Green—caregiver).

### Resources—Support and Stability

Resources relates to the external supports that contribute to a couple's resilience in living with dementia. The support of others was imperative to be able maintain resilience, as a caregiver stated “you need people, you need family or someone who cares” (Ms. Williams—caregiver). People providing support included family (talking about their daughter); “she's been very supportive because she comes round and you know she makes sure that we're alright and everything” (Mr. Roberts—PLwD), friends; “I can't express enough about friends, you need… you must have a decent base of friends” (Mr. Smith—PLwD), neighbors; “the neighbors are very good, and if anything was wrong they're here” (Mrs. Brown—caregiver) and even pets; “I'd be totally lost without my dogs, I couldn't be without one” (Mrs. Thompson—caregiver). However, couples highlighted the importance of also not becoming too reliant on others, with one caregiver stating “you just rely on each other and you just get on with it, don't you, you don't rely on anybody else” (Mrs. Davies—caregiver).

Couples also valued the support of professionals in the maintenance of resilience. This included healthcare professionals from whom couples valued consistency and collaboration, which Mr. Evans stated was beneficial as “consequently I think there isn't anything that we experience we wouldn't tell him [GP] is there?” (Mr. Evans—PLwD). Other couples sought support from dementia support groups, enabling PLwD in the dyad to meet other people, learn about dementia, whilst also providing a routine for the couple and a different perspective on life with dementia. However, dementia support groups were not attended by all, as one caregiver stated it was “people just moaning” (Mrs. Taylor—caregiver), and also PLwD reportedly worrying that it showed what the future might hold for them; “he doesn't want to go, because there will be people there with advanced dementia” (Mrs. Davies—caregiver).

Financial stability was helpful for couples to manage dementia, enabling them to afford to make adaptations to their homes in order to maintain a similar kind of life as before and go on holidays to give themselves a break. Having financial stability also provided couples with peace of mind that should the PLwD need to go into a care home, they would be able to afford one which felt like home. For other couples, the benefits of finances were less about affording the luxuries and more about affording the basics to continue the life they enjoyed, with a caregiver stating “if I was cold or uncomfortable or hungry all the time I'd be in a bad temper” (Ms. Williams—caregiver).

### Emergent Theory

For couples living with dementia, resilience is a process of maintenance, related to the ability to adapt together in order to continue to live a good shared life. Couples utilized two perspectives to remain resilient; a positive outlook and fighting the dementia. Their sense of couplehood served as evidence to them of their resilience but was also recognized as contributing to their resilience. Shared resilience enabled each partner to maintain and develop their individual resilience, whilst the development and maintenance of individual resilience in turn helped develop and grow their shared resilience. As such shared resilience was equally valued by and valuable for both partners. This reciprocal process resulted in the continual development of resilience throughout the couples' lives together, which continued through the journey with dementia. Shared resilience ebbed and flowed depending on the amount the couple had to cope with on a day-to-day basis and the support they received. Shared resilience therefore was dynamic and continued to develop over time, enabling couples to continue living well despite the challenges of dementia.

## Discussion

A key aim of the study was to develop an understanding of what resilience meant to couples living with dementia. However, perhaps understandably, couples often found resilience difficult to define. This is consistent with previous research that has asked caregivers of PLwD about resilience (24). Yet despite the difficulties defining resilience, all couples were able to talk about what it meant to them and how it was experienced, describing resilience fundamentally as their ability to continue living a normal life together. This “everyday resilience” diverges from more prominent definitions of resilience as recovering, bouncing back (3) or flourishing (25). Findings from our study concur with the idea that resilience is an “ordinary magic” (26), in which people adapt and change to cope with the everyday difficulties they face. This is consistent with the findings that in later life (14) and in dementia (8), resilience is a process of continuity and perceived maintenance of functioning, with people adapting how they do things, not what they do.

A central finding from this study is the importance of the relationship in maintaining and developing shared resilience whilst living with dementia. When couples considered their shared sense of resilience, they intuitively drew upon their sense of couplehood and the shared strategies they used to continue to lead what they considered to be a “normal life.” This emphasizes the importance of dyadic research in developing a clearer understanding of the experience of resilience. Ideas of family resilience, in which the relationship of the family is vital for the development of a shared sense of resilience between its members (27), and relational resilience are not new. Indeed, the value of shared resilience has been recognized in other chronic illnesses (28). Furthering this dyadic research in dementia is essential to learn more about reciprocity in relationships and its role in supporting living well.

Couples took two different perspectives in order to maintain a normal life in the face of dementia: positivity and fighting. This reflects Clare's (29) finding that people adjust to early stage dementia by developing a “fighting spirit,” in which they face the threats of dementia head on, alongside “holding on” and compensating, trying to preserve their sense of self. Whilst Clare's (29) study focused on individuals in the early stage of Alzheimer's dementia, our findings capture the experiences of dyads in both the early and mid-stages of dementia. This suggests that the perspectives of positivity and fighting the dementia can be maintained along the journey of dementia as symptoms progress, but also can be held together as a couple. Within the current study most couples utilized both perspectives flexibly in order to maintain a normal life. However, older couples and those who had been living with dementia for longer tended to focus on remaining positive (i.e., using humor) rather than maintaining a fighting spirit, possibly due to it becoming harder to “fight” dementia as it progresses. In addition to this, it may be that remaining positive becomes an important way to experience positive emotions whilst also preserving a sense of mutuality in relationships (i.e., staying positive together) as dementia progresses.

The finding that resilience is dynamic and develops over time resonates with perspectives which define resilience as a cyclical process as opposed to a trait which people either do or do not possess (30). This has also been demonstrated in resilience research with caregivers in dementia (24). Our emergent theory therefore presents the development and maintenance of shared and individual resilience as a cyclical process, something which Windle and Bennett's (14) framework has been criticized for lacking in relation to caregivers in dementia (24). Despite this critique, the resources enabling shared resilience identified within this study could be divided into individual, community, and society resources, the taxonomy Windle and Bennett (14) outline within their framework. Findings from this study therefore provide evidence that couples living with dementia may utilize similar resources to remain resilient as caregivers of older adults.

This is an exploratory study, informing emergent theory. However we may consider some tentative suggestions for clinical practice at this early stage. The integration into practice of the consistent and specific assessment of resilience to inform and guide well-targeted plans for intervention is the next logical step. Asking couples what they value in their shared life together, what has got them through difficult times in terms of their shared experiences and capabilities may lead to the identification of asserts and resources that can serve as the foundation for clinical intervention. It is important to talk with dyads about the “ordinary magic” of resilience to avoid the “tyranny of the positives” (31) and any mandating of what a good life with dementia looks like. Couples should expect resilience to ebb and flow and a shared life review may help couples to recognize this lifelong pattern of resilience. Clinicians should not overlook the value of small actions to promote resilience; demonstrating acts of love, planning for financial stability and getting affairs in order and knowing what support is available when needed and finding time alone.

## Limitations

A strength of this research lies in its dyadic approach; taking both perspectives into account provides a better understanding of the changes and processes taking place within relationships as dementia progresses. Interviewing dyads together generates a comprehensive insight into the dyadic interplay but it also raises challenges in relation to social desirability of responses and openness in front of the other partner. As such, a key procedural issue is managing the conversation during data collection so as to ensure that both members of the dyad are equally heard. It might be expected that one member of the couple, usually the carer, might dominate the conversation and talk for the person with dementia. This may have been the case historically or it might be the result of a carer becoming accustomed to answering on behalf of the person living with dementia.

These issues reflect the influence of relationship factors and dynamics on how dyads respond to and talk about their experiences of dementia. Prior relationship quality in dementia impacts on emerging relationship style as dementia progresses; dyads able to maintain high levels of mutuality [positive, reciprocal interactions; see (32)] in their relationship do so by focusing on continuation of roles and preservation of identity and/or through a deliberate focus on reciprocation. In contrast, for dyads (couples) with low prior relationship quality, the style of the caring relationship might be based on detachment and/or a sense of duty, which is likely to be associated with lowered well-being for both members [see (33)]. Similarly, couples' efforts to nurture their sense of togetherness and resilience in dementia run in parallel with how they negotiate changes in role and power, preserve identity, and maintain empathic attunement [see (11)].

In recognition of these issues, efforts were made during interviewing to ensure questions were directed toward and opened to both members of the dyad, and whilst in some interviews carers did more of the talking, this was certainly not always the case. At the same time, prior and current relationship dynamics were not directly investigated. This leaves open the possibility that the way couples described their experiences of shared resilience was influenced by long-standing relationship quality and/or shifts in role, power, and empathy. Key differences between couples/dyads in these areas and the impact of these differences on experiences of shared resilience should be the focus of further research.

In order to reach saturation we purposively sampled volunteer participants. However, it could be argued that a limitation of the study was the lack of diversity amongst participants. According to the social graces model (34), diversity is a multi-faceted concept that includes gender, geography, race, ethnicity, and sexual orientation. When considering gender, the majority of caregivers involved in the current study were female, whilst the majority of PLwD were male. Research has found there are gender differences in the experience of providing care for PLwD (35) and future research could therefore aim to recruit gender balanced samples. Couples in this study were also recruited from a small regional area of the UK, with all of the participants identifying as White British. Furthermore, participants were not asked about the perceived impact of their ethnicity or geography. As such, the generalizability of our findings and emergent theory may be limited. Finally, in relation to sexual orientation, each of the couples interviewed were in heterosexual relationships. McParland and Camic (36) found evidence that dementia is experienced differently by same sex couples. As such, future research should also include same sex partnerships or aim to expand our understanding of how differences in experiences according to sexual orientation are experienced.

In addition to limitations relating to diversity, this research only offers an insight into shared resilience within romantic relationships. There are a number of other caregiving relationships in which shared resilience may be developed and maintained, such as parental and sibling relationships or friendships. Future research could therefore consider how different types of care relationships may foster shared resilience in dementia.

During the interviews, couples reflected on whether, as they continue through the journey of dementia, their resilience will change. Resilience research could therefore benefit from either being longitudinal to understand how a shared sense of resilience might change over time or include couples who have been living with dementia for longer.

## Conclusions

Living well has been a rhetoric in dementia care for some time; we need to develop our understanding of how people live well and what strengths they draw up. Key to this is recognizing that people do not flourish in isolation and that couples will have shared strengths and resources. This study is important in its dyadic focus to enable further insight into our understanding of resilience and living well with dementia. In contrast to models of resilience which emphasize bouncing back or flourishing in the face of adversity, the couples in this study described resilience as continuing with a “normal” life notwithstanding the impact of dementia. Couples described a shared resilience, which enabled them to maintain their couplehood—that is their shared sense of togetherness and the reciprocity in their relationship. This sense of couplehood in turn provided another source of resilience. Our findings emphasize the value of dyadic research and interventions in developing our understating of living well with dementia and how we can support couples to maintain and enhance their resilience.

## Data Availability Statement

The raw datasets will not be made publicly available due to ethical constraints. Requests can be made and taken under consideration by contacting EW, e.wolverson@hull.ac.uk.

## Ethics Statement

The studies involving human participants were reviewed and approved by the London—Riverside Research Ethics Committee and the Health and Research Authority (Approval No. 17/LO/1121). The patients/participants provided their written informed consent to participate in this study.

## Author Contributions

LC, EW, and CC designed the study collaboratively. LC collected the data and led the data analysis, with EW and CC helping in refining and developing themes. LC drafted the initial manuscript. EW and CC revised and edited the manuscript to help prepare for submission.

## Conflict of Interest

The authors declare that the research was conducted in the absence of any commercial or financial relationships that could be construed as a potential conflict of interest.
